# *In Vitro* Matured Oocytes Are More Susceptible than *In Vivo* Matured Oocytes to Mock ICSI Induced Functional and Genetic Changes

**DOI:** 10.1371/journal.pone.0119735

**Published:** 2015-03-18

**Authors:** Shubhashree Uppangala, Shilly Dhiman, Sujit Raj Salian, Vikram Jeet Singh, Guruprasad Kalthur, Satish Kumar Adiga

**Affiliations:** Division of Clinical Embryology, Department of Obstetrics & Gynecology, Kasturba Medical College, Manipal University, Manipal-576 104, India; University of Crete, GREECE

## Abstract

**Background:**

Concerns regarding the safety of ICSI have been intensified recently due to increased risk of birth defects in ICSI born children. Although fertilization rate is significantly higher in ICSI cycles, studies have failed to demonstrate the benefits of ICSI in improving the pregnancy rate. Poor technical skill, and suboptimal *in vitro* conditions may account for the ICSI results however, there is no report on the effects of oocyte manipulations on the ICSI outcome.

**Objective:**

The present study elucidates the influence of mock ICSI on the functional and genetic integrity of the mouse oocytes.

**Methods:**

Reactive Oxygen Species (ROS) level, mitochondrial status, and phosphorylation of H2AX were assessed in the *in vivo* matured and IVM oocytes subjected to mock ICSI.

**Results:**

A significant increase in ROS level was observed in both *in vivo* matured and IVM oocytes subjected to mock ICSI (P<0.05-0.001) whereas unique mitochondrial distribution pattern was found only in IVM oocytes (P<0.01-0.001). Importantly, differential H2AX phosphorylation was observed in both *in vivo* matured and IVM oocytes subjected to mock ICSI (P <0.001).

**Conclusion:**

The data from this study suggests that mock ICSI can alter genetic and functional integrity in oocytes and IVM oocytes are more vulnerable to mock ICSI induced changes.

## Introduction

Intracytoplasmic sperm injection (ICSI) has become the treatment of choice for several infertility disorders; however, concerns regarding the safety of this technique have been intensified recently due to increased risk of birth defects in ICSI born children [1–4]. Although fertilization rate is significantly higher in ICSI cycles, several studies have failed to demonstrate the benefits of ICSI in improving the pregnancy rate [5–6]; possibly due to poor developmental competence of the embryos [7], compromised implantation [8] and post-implantation developmental potential [9]. These observations prompt more basic research to assess the safety of ICSI [10–11].

Several sperm mediated factors can affect the ICSI outcome [12–15]. Incorporation of the acrosome into the oocyte during ICSI has shown to be potentially hazardous to embryo development [16]. Similarly, polyvinylpyrrolidone (PVP) which is being used successfully in human ICSI for sperm immobilization has shown adverse effects on gametes and embryos [17–20]. On the other hand, the poor technical skill, and suboptimal *in vitro* conditions may also be detrimental to the embryonic development and impair ICSI outcome [21–22]. However, there is no report on the effect of oocyte manipulations on the ICSI outcome.

Although ICSI has been successful in many other species, the ability of oocytes to tolerate microinjection process is related to the species [23] possibly due to differences in oocyte ultrastructure. Mouse is considered as a model organism to study mammalian fertilization. However, the ability to fertilize mouse oocytes by conventional ICSI is difficult [24]. Due to increased sensitivity of mouse oocytes to micromanipulation and also ethical restrictions in using human oocytes, we have used mouse model to elucidate the effects of *in situ* manipulations such as ooplasma aspiration and microinjection on the functional and genetic integrity of the oocytes. In order to rule out the sperm mediated effects, only mock injection was performed without sperm involvement. In addition to *in vivo* matured oocytes, *in vitro* matured (IVM) oocytes were also used since IVM process makes the oocyte more vulnerable to embryological interventions [25]. Injected oocytes were activated parthenogenetically to elucidate the influence of mock ICSI on the functional and genetic integrity of the oocytes.

## Materials and Methods

### Animals

Eight weeks old healthy Swiss albino female mice (N = 62) were used in this study. The animals were maintained under the controlled conditions of temperature (23±2°C) and light (12h light/dark cycles) with standard diet and water *ad libitum*. Institutional Ethical Committee of Kasturba Medical College, Manipal University (IAEC/KMC/03/2011–2012) approval was taken prior to the commencement of the study.

### Superovulation and MII oocytes retrieval

Female mice were superovulated by intraperitoneal injection (5IU) of pregnant mare serum gonadotropin (PMSG, Cat No. G4877 Sigma Aldrich, USA) followed by the administration of 10IU of human chorionic gonadotropin (hCG, Eutrig-HP) after 48h. Animals were euthanized 12h post hCG by cervical dislocation and oocyte cumulus complexes (OCC) were released from the oviduct into the pre-warmed M2 medium. OCCs were exposed to 0.1% hyaluronidase (Cat. No. 4272 Sigma Aldrich, USA) for 30 seconds and oocytes were washed in M16 medium and then incubated in M2 medium at 37°C under 5% CO_2_.

### GV oocyte collection and *In Vitro* maturation

Animals were euthanized and the ovaries were collected in pre-warmed M2 medium. The cortical region of the ovaries was gently teased using fine needles and the Germinal vesicle (GV) oocytes were released from the secondary/tertiary follicles. The GV oocytes were further subjected to IVM by culturing in 20μL of Dulbecco’s Modified Eagle’s Medium (DMEM, Cat. No. D5648, Sigma Aldrich, USA) supplemented with 1% Non-essential α-amino acids (Cat. No. M7145, Sigma Aldrich, USA), 1% Insulin-Transferrin-Selenium (ITS, Cat. No. 51500–056 Gibco, USA), 0.05% pyruvate and 0.3% BSA overlaid with oil. Oocytes were incubated at 37°C in 5% CO_2_ for 24h and then assessed for nuclear maturity. Oocytes with polar body were considered as mature (metaphase II).

### Mock ICSI

Both *in vivo* matured and IVM oocytes were randomly divided into three groups. Two sets of controls (Standard control: oocytes incubated at 37°C and 5% CO_2_; ICSI control: oocytes kept in the ICSI dish without mock injection) and ICSI group (oocytes kept in the ICSI dish subjected to mock injection) were included in this study. ICSI dish had about ten micro droplets of 3.5μL M16 medium where single mature oocytes were placed in each droplet. Approximately 10μL of PVP based immobilization medium (PVP Clinical Grade, Cat # 10900000A, Origio, Medicult) was placed adjacent to oocyte droplets and the dish was overlaid with mineral oil. Mock injection was carried out using an inverted microscope (Olympus IX71, Tokyo, Japan) at a magnification of 350X, on a heated stage (MATS-USMZSS, Thokai Hit, Japan) maintained at 37°C. Two micromanipulators (Narishige, Tokyo, Japan) were mounted on the microscope that allowed the micro-movements of two needles on a three-dimensional plane. Briefly, oocytes were positioned by keeping the polar body at 6 o’ clock position and held with gentle suction using holding pipette. Injection needle was first equilibrated in PVP followed by zona penetration, then ooplasm was aspirated and released without the deposition of sperm into the oocyte. A time period of 30 min was maintained for the entire procedure and after 30 min, oocytes were further processed for different parameters as described in the subsequent sections. Mock ICSI in both the groups was performed by a single person. Care was taken while aspirating the cytoplasm to the fixed level.

### Measurement of Reactive Oxygen Species

The intracellular levels of reactive oxygen species (ROS) in oocytes was measured by 2′',7′-dichlorodihydrofluorescein diacetate (DCHFDA, Cat No. D6883, Sigma Aldrich, USA) fluorescence assay as described earlier [26]. Thirty minutes after mock injection, oocytes in three study groups were incubated separately in 10μM DCHFDA prepared in M16 medium for 30min at 37°C and 5% CO_2_. Oocytes were then washed to remove surface-bound dye before being mounted between a slide and coverslip. Fluorescence emissions of oocytes were recorded using a fluorescent microscope (Imager-A1, Zeiss, Gottingen, Germany) under UV light with filter at 405–435nm. Appropriate positive and negative controls were used in each experiment. Images were acquired and the intracellular ROS levels were quantified using Q Capture software (Media Cybernetics Inc., USA).

### Assessment of mitochondrial distribution

The mitochondrial distribution pattern was assessed in both *in vivo* matured IVM oocytes as previously described [27] with minor modification. Briefly, oocytes were incubated in M16 medium containing 10μg/mL of Rhodamine 123 (Cat No. R8004, Sigma Aldrich, USA) for 20min at 37°C and 5% CO_2_. After wash, the oocytes were transferred onto a glass slide containing about 20μL of fluorescent mounting medium (Cat. No. S3023, Dako, USA), carefully observed under UV light (405–435nm) using a fluorescent microscope (Imager-A1, Zeiss, Gottingen, Germany) and the images were acquired using Q Capture imaging software (Media Cybernetics Inc. USA). The patterns of mitochondrial distribution were classified according to Liu *et al*. [28] as follows: i) uniform distribution: mitochondria spread uniformly all over the cytoplasm ii) aggregated distribution: mitochondria distributed in irregular clumps and iii) peripheral distribution: mitochondria localized in the periphery of the oocytes.

### Measurement of mitochondrial activity

The mitochondrial activity in mock injected IVM oocytes was determined as described earlier [29] with minor modifications. Briefly, the oocytes were exposed to 1μg/mL 5,5′,6,6′-tetrachloro-1,1′,3,3′-tetraethylbenzimidazolylcarbocyanine iodide JC-1 (Cat. No. T3168, Molecular probes, Life technologies, USA), for 30 min at 37°C followed by wash in DMEM medium supplemented with 1mg/mL of bovine serum albumin. JC-1 monomers and JC-1 aggregates were detected in a using a fluorescence microscope (Imager-A1, Zeiss, Gottingen, Germany). The ratio of orange and green fluorescence was calculated using ImageJ software (National Institute of Health, Bethesda, Maryland, USA).

### Parthenogenetic activation

The oocytes were subjected to parthenogenetic activation as described by Ma *et al*. [30] with minor modifications. Briefly oocytes were incubated in 20μL droplet of M16 medium (Ca^++^ Mg^++^ free) containing 10mM SrCl_2_ at 37°C in 5% CO_2_ for 3h and then washed three-times in M16 medium. Pronuclear formation was scored to determine the rate of activation and only activated oocytes were used for immunofluorescence.

### γ-H2AX detection

Immunofluorescence detection of γ-H2AX was performed according to the previously described method [31] with minor modifications. Oocytes were washed with phosphate buffered saline (PBS) and fixed with 4% paraformaldehyde in PBS for 30 min and then permeabilized for 30 min at room temperature with PBS containing 0.1% (V/V) Triton X-100 and 0.5% bovine serum albumin. They were then stained with anti-phospho-Histone H2AX antibody (1:20, Cat. No. 05–636; Upstate Biotechnology, Billerica, MA, USA) incubated overnight at 4°C followed by treatment with secondary antibody conjugated with fluorescein isothiocyanate (FITC) (Goat anti-mouse IgG, Cat. No. sc-2010, 1:50 dilution, Santa Cruz Biotechnology, Dallas, TX, USA) for 1h at 37°C. The immunostained oocytes were washed with PBS containing 0.1% triton-X, counterstained with 4',6-Diamidino-2-phenylindole (DAPI), and observed under fluorescent microscope. Immunofluorescent signals were recorded using a fluorescent microscope (Imager-A1, Zeiss, Gottingen Germany) equipped with Q-imaging Micropublisher 5.0 RTV digital camera (Qimaging Surrey, BC, Canada). The number of foci bearing oocytes and the average number of foci per cell were calculated from a minimum of 30 oocytes per data point. In addition, the foci were categorized as small (< 1μm diameter), and large (> 1μm diameter) in each group.

### Statistical analysis

The data represents either mean and standard error (Mean ± SEM) or percentage of the values. The statistical significance level was calculated using Chi square test, Fischer’s exact test, Mann- Whitney U test and Kruskal-Wallis test followed by Dunn post-test for multiple comparisons using GraphPAD Instat software (Graphpad Inc., La Jolla, CA, USA). The graphs were plotted using Origin 6.0 (Origin Lab Corporation, Northampton, MA, USA).

## Results

### Mock ICSI increased ROS level in both *In Vivo* matured and IVM oocytes

**Fig 1:**
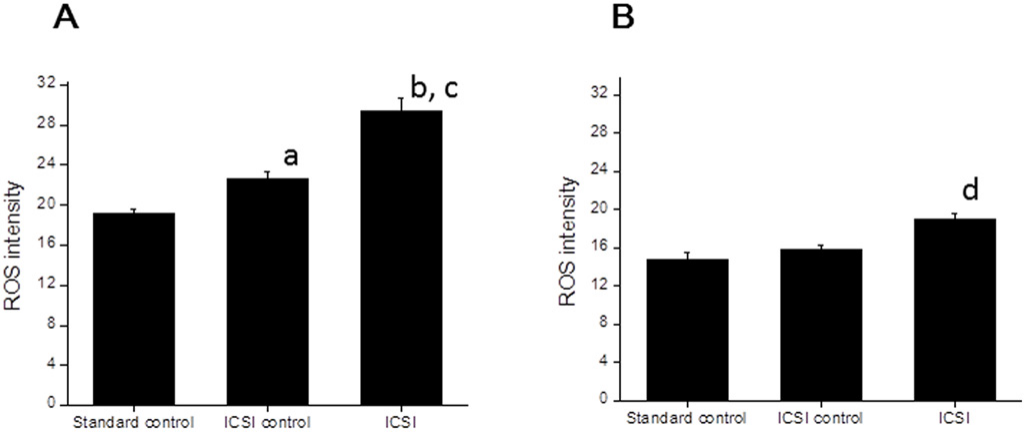
Effect of mock injection on Reactive oxygen species level in *in vivo* and *in vitro* matured murine oocytes assessed using 2′,7′-dichlorodihydrofluorescein diacetate (DCHFDA) staining. A. Oocytes retrieved from superovulated mice, subjected to mock ICSI were assessed for ROS production. The relative ROS intensity in standard control (N = 19), ICSI control (N = 22) and ICSI group (N = 20), was determined. The error bars represent the corresponding SEM (Mean ± SEM). ^a^P <0.05: Standard control Vs ICSI control, ^b^P <0.01: Standard control Vs ICSI group, ^c^P < 0.001: ICSI control Vs ICSI group. B. *In vitro* matured metaphase II oocytes, subjected to mock ICSI were assessed for ROS production. The relative ROS intensity in standard control (N = 28), ICSI control (N = 25) and ICSI group (N = 26), was determined (Mean ± SEM). ^d^P < 0.001: Standard control Vs ICSI group and ICSI control Vs ICSI group.

A total of 61 *in vivo* matured and 79 IVM oocytes were included in order to assess the effect of mock ICSI on intracellular levels of reactive oxygen species. The ROS level in oocytes was quantified in all three groups as described in the materials and methods. The data on baseline ROS level was recorded in the oocytes from standard control group whereas ICSI control group served as an internal control. The *in vivo* matured oocytes in both ICSI control and ICSI group had significantly higher levels of ROS in comparison to the standard control (P < 0.05, P < 0.001 respectively, Fig. 1A). Further, the ICSI group had significantly higher level of ROS compared to ICSI control (P < 0.01, Fig. 1A). In contrast to *in vivo* matured oocytes, IVM oocytes did not show any significant difference between standard control and ICSI control. However, ICSI group had significantly higher level of ROS in comparison to standard control and ICSI control (P <0.001) (Fig. 1B). Interestingly, *in vivo* matured oocytes in ICSI group had almost 1.5 fold increase in ROS level in comparison to corresponding group in IVM (P < 0.0001).

### IVM oocytes were more susceptible to mock ICSI induced changes in mitochondria

**Fig 2:**
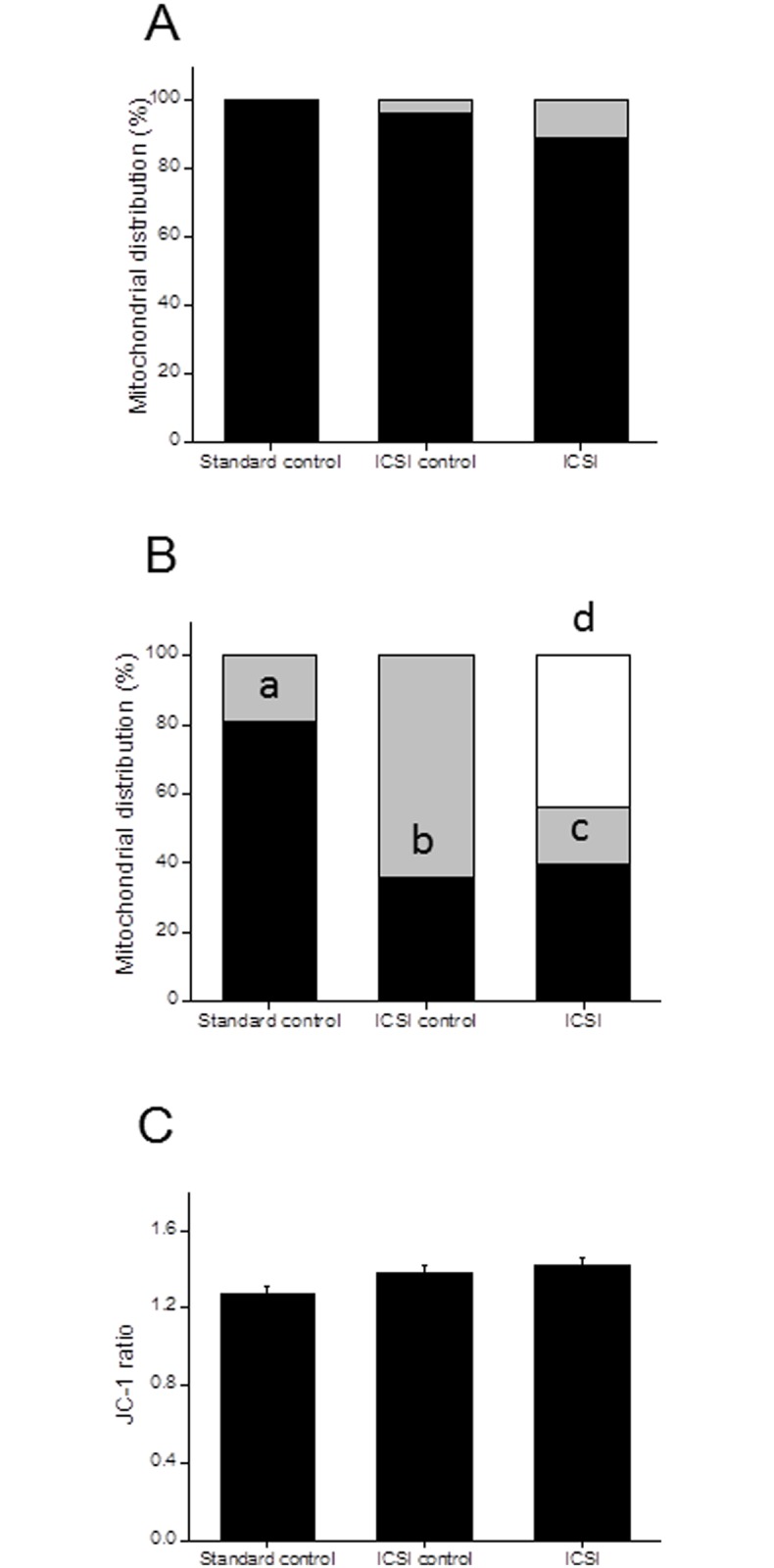
Mitochondrial distribution and activity as measured by Rhodamine 123 and JC1 staining of oocytes. A. Oocytes retrieved from superovulated mice, subjected to mock ICSI were assessed for mitochondrial distribution. The percentage of oocytes displaying uniform (closed bar); and aggregated (grey bar) distribution in standard control (N = 28), ICSI control (N = 49) and ICSI group (N = 36), was determined. B. *In vitro* matured metaphase II oocytes, subjected to mock ICSI were evaluated for mitochondrial distribution. The percentage of oocytes displaying uniform (closed bar); aggregated (grey bar) and peripheral (open bar) distribution in standard control (N = 33), ICSI control (N = 33) and ICSI group (N = 32), was determined. ^a^P <0.05: Uniform distribution pattern in standard control of figure A Vs Standard control in figure B. ^b^P < 0.001: Standard control Vs ICSI control. ^c^P < 0.01: Standard control Vs ICSI group. ^d^P < 0.0001: percentage of oocytes displaying peripheral distribution in ICSI group with other two groups. C. Mitochondrial activity as measured by the JC1 ratio in IVM oocytes in standard control (N = 81); ICSI control (N = 69) and ICSI group (N = 68). Please note that difference were not significant.

Mitochondrial distribution pattern was measured in 113 *in vivo* matured and 98 IVM oocytes. Importantly, mitochondrial distribution pattern did not vary significantly between three groups of *in vivo* matured oocytes and majority of the oocytes had uniform distribution pattern (Fig. 2A). In contrast, a significant decline in uniform mitochondrial distribution pattern was observed in IVM derived standard control oocytes in comparison to that of *in vivo* matured one (P <0.05). On the other hand, both ICSI control and ICSI groups had significantly lower number of oocytes displaying uniform pattern in comparison to standard control (P < 0.001 and P < 0.01 respectively) (Fig. 2B). Interestingly, ICSI group had approximately 44% of oocytes with peripheral mitochondrial pattern which was not observed in any other groups of *in vivo* matured and IVM oocytes (P < 0.0001).

Since IVM oocytes subjected to mock ICSI displayed a unique peripheral distribution, oocytes were subjected to JC-1 staining to understand the mitochondrial activity. JC-1 aggregates forming multimers that fluoresce orange in active mitochondria and green from JC-1 monomers in inactive mitochondria. Though, we observed a moderate increase in the ratio, the difference was not statistically significant. These observations suggest that altered distribution pattern observed in mock injected oocytes may not affect mitochondrial activity significantly (Fig. 2C).

### Parthenogenetic activation rate was unaffected by mock ICSI

To assess whether mock injection affects the developmental competence of oocytes, oocytes from all the three groups were parthenogenetically activated using SrCl_2._ The activation rate in the *in vivo* matured oocytes was not statistically significant between standard control (67%), ICSI control (81%) and ICSI (83%). Similarly, IVM oocytes did not show statistically significant difference between three groups (standard control: 38%; ICSI control: 44% and ICSI: 37%) (Table 1). However, overall activation rate in IVM oocytes was significantly lower than *in vivo* matured oocytes in the corresponding subgroups (P < 0.01).

**Table 1:** Parthenogenetic activation rate across study groups.

Study group	In vivo MII oocytes (N)	Activated oocytes (%)	In vitro matured oocytes (N)	Activated oocytes (%)
Standard control	49	33 (67)	81	31 (38)
ICSI control	49	40 (81)	81	36 (44)
ICSI	49	41(83)	81	30 (37)

### Phosphorylated H2AX in oocytes subjected to mock injection

**Fig 3:**
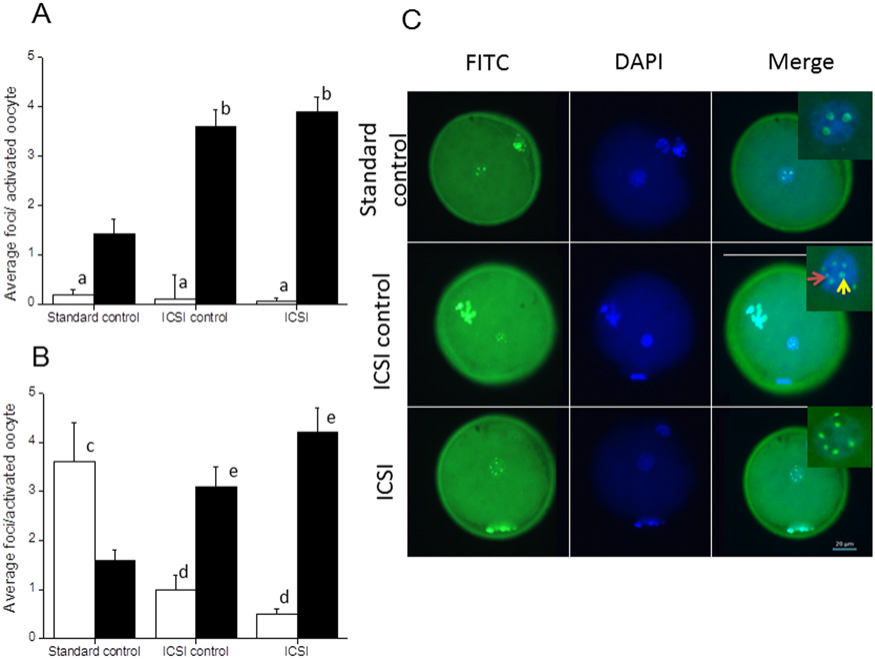
γ-H2AX immunolocalization in GV oocytes. (A) Oocytes retrieved from superovulated mice, subjected to mock ICSI were assessed for γ-H2AX foci. The average number of foci bearing oocytes from standard control (N = 33), ICSI control (N = 40) and ICSI group (N = 41) from a total of 19 animals were evaluated. ^a^P < 0.0001 between small foci (open bar) and large foci (closed bar) in corresponding groups. ^b^P < 0.001: standard control Vs ICSI control and ICSI group. B. *In vitro* matured metaphase II oocytes, subjected to mock ICSI were activated parthenogenetically to assess γ-H2AX foci. The average number of foci bearing oocytes from standard control (N = 31), ICSI control (N = 36) and ICSI group (N = 30) from a total of 14 animals were evaluated. ^c^P < 0.0001: Small foci in *in vitro* matured Vs *in vivo* matured oocytes. ^d^P < 0.001: Small foci in ICSI control and ICSI groups Vs standard control. ^e^P <0.001: Large foci in ICSI control and ICSI groups Vs standard control. (C) Representative images of γ-H2AX foci in three groups (enlarged foci are shown in the insert, red arrow shows small focus and yellow arrow shows large focus). The scale bar applies to all nine micrographs.

A significant increase in the ROS level and altered mitochondrial distribution pattern in mock injected oocytes prompted us to look into the prevalence of DNA double strand breaks (DSB’s) and the repair process in *in vivo* matured and IVM oocytes subjected to mock injection. To address this, parthenogenetically activated oocytes were immunostained to detect H2AX phosphorylation. Since γ-H2AX foci size reflects specific biological response, the number of small (< 1μm diameter) and large foci (> 1μm diameter) were analyzed in each group. The number of small foci was significantly lower than large foci in all three groups of *in vivo* matured oocytes (P <0.0001). The number of large foci in *in vivo* matured ICSI control and ICSI groups was almost 2.5 fold higher than standard control and the differences were statistically significant (P <0.001). The oocytes in ICSI control and ICSI groups had comparable number of large foci hence the difference was not statistically significant (Fig. 3A). On the other hand, IVM derived oocytes in standard control had almost 18 fold higher number of small foci in comparison to corresponding group in *in vivo* matured oocytes (P <0.0001). Conversely, the number of large foci in IVM groups was comparable to *in vivo* matured groups. On the other hand, the number of small foci was significantly lower in both ICSI control and ICSI groups in comparison to standard control of IVM oocytes (P < 0.001). In contrast to small foci, the number of large foci in ICSI control and ICSI groups were significantly higher than standard control in IVM oocytes (P < 0.001) (Fig. 3B,C).

## Discussion

We present here a study using a mouse model to test the impact of oocyte manipulation by mock ICSI on the functional and genetic integrity of the oocytes. To our knowledge, this is the first time significant alterations in ROS production and mitochondrial distribution pattern have been observed in the oocytes subjected to ICSI conditions. Though mitochondrial pattern was unaffected in *in vivo* matured oocytes, it is interesting to note that, mock ICSI resulted in a unique peripheral mitochondrial distribution in *in vitro* matured oocytes. Importantly, highly significant, distinct H2AX phosphorylation triggered by mock ICSI was observed in IVM oocytes.

To mimic clinical ICSI, a modified technique close to clinical situation was employed in mouse oocytes without sperm deposition in the ooplasm. The overall success in terms of degeneration rate following manipulation, oocyte granulation and fragmentation was not significantly different from control groups (data not shown) indicating that there was no immediate negative effect of manipulations on overall efficiency.

ROS that are produced in measurable quantities by every aerobic system were considered toxic to living cells [32]. In the present study, apart from *in vivo* matured mouse oocytes, GV oocytes were cultured *in vitro* to attain maturity and after 24h of maturation, ROS level was assessed in oocytes post mock injection. It has been shown that IVM derived oocytes were highly susceptible to oxidative stress [33]; hence we speculated increased ROS level in IVM oocytes. To our surprise, the baseline ROS level in IVM oocytes was significantly lower than *in vivo* matured oocytes. Since ovarian production of ROS is transiently triggered by LH [34], lack of LH stimuli in IVM system possibly resulted in a lower level of ROS in these oocytes. It has been suggested that certain amount of ROS is essential for the successful fertilization process [35]. In case of sperm-activated oocytes, ROS peaks are associated with fertilization events such as sperm penetration and sperm head decondensation [36]. However, our data suggest that increase in ROS level is independent of sperm deposition but may be related to mechanical stress induced during mock injection process since both *in vivo* matured and IVM oocytes subjected to mock injection had significantly higher level of ROS compared to standard and ICSI controls.

Since mitochondria are the sources and targets of ROS, we next examined the mitochondrial distribution pattern in mock injected oocytes. The patterns of mitochondrial distribution in mock injected IVM oocytes were distinct from those oocytes matured *in vivo*, suggesting that mitochondrial organization is impaired following mock injection. The localization of mitochondria in the oocyte during maturation is strictly regulated [37] and it has been suggested that changes in mitochondrial organization is a reliable indicator of oocyte capacity to sustain embryonic development [38]. In addition, the migration of mitochondria towards center and its uniform distribution is believed to be an indication of cytoplasmic maturation [28]. Interestingly, IVM oocytes, which have lower developmental capacity than those matured *in vivo*, display reduced mitochondrial transport to the central region which is suggested to be associated with a reduced cytoplasmic microtubule network [39–40]. Though, approximately 80% of IVM oocytes in the standard control displayed uniform distribution pattern, it is important to note that a significant number of oocytes displayed aggregated distribution when exposed to *in vitro* conditions for a period of 30 minutes. Importantly, mock injection has resulted in peripheral localization of mitochondria in about 44% oocytes possibly due to cytoskeletal disruption induced by ooplasm aspiration which could impact the distribution of mitochondria [41–42]. To understand the impact of peripheral distribution pattern observed in mock ICSI group on the mitochondrial integrity, JC1 staining strategy which was previously been used to measure the mitochondrial activity in mouse oocytes [43] was employed. However, alteration in the JC1 ratio did not correlate the change in distribution pattern suggesting that mitochondrial distribution but not activity is affected in mock injected IVM oocytes.

Since normal mitochondrial function is required for the formation of oocyte spindles, we next examined the functional ability of the oocytes subjected to mock ICSI. To avoid the sperm mediated effects in evaluating ICSI induced changes in the oocytes, our study used parthenogenetic activation for assessing the activation potential. The analysis of parthenogenetic activation potential in both *in vivo* matured and IVM derived metaphase II oocytes did not reveal significant differences between mock injected and control groups though overall activation rate was significantly lower in IVM oocytes.

Increased ROS level and altered mitochondrial distribution in mock injected oocytes may render oocyte DNA to ROS-induced DNA damage. Earlier study has shown that ICSI derived mouse embryos had abnormal chromosomal conformation during segregation at first mitotic division and consequently formed micronuclei like structures at the two cell stage [44]. Micronuclei in embryos are usually originated in response to genetic insult and increased micronuclei frequency is suggestive of embryonic genetic instability [45–46]. Extensive chromatin remodeling occurs during the process of pronuclear formation and this appears to be the determining factor for the normal onset of gene expression [47]. Hence, we assessed the phosphorylation of H2AX in the pronuclei of activated oocytes derived from mock injection. H2AX histone characteristically undergoes phosphorylation at serine 139 in response to DNA damage and DNA DSB’s in somatic cells [48–50]. Immunolocalization of γ-H2AX in activated pronucleus showed distinct patterns of foci (Fig. 3C). The size of the foci is known to reflect specific biological response hence we categorized the foci into small and large types based on the diameter of the foci. Small γ-H2AX foci are associated with cell cycle regulation and mitosis whereas larger foci are suggestive of recruitment of DNA repair proteins or accumulation of DNA DSB aggregates [51–52]. *In vivo* matured oocytes had relatively few small foci in all three groups. On the other hand, both ICSI control and ICSI group had similar number of large foci though the incidence was significantly higher than the *in vivo* matured standard control. These observations suggest that DSBs in *in vivo* matured oocytes were induced even without mock injection but possibly by exposing the oocytes to *in vitro* conditions for 30 min. Several factors may account for these changes. It has been shown that HEPES buffering system present in the culture medium can be detrimental to the embryos if used during ICSI [22]. In addition, pH changes in ICSI medium [53], temperature variations [54], light used in the microscope [55] may influence the functional and genetic integrity of the oocytes. Interestingly, the number of small foci in IVM oocytes was significantly higher from *in vivo* matured oocytes especially baseline level of small foci in IVM oocytes was almost 18 fold higher than corresponding group in *in vivo* matured oocytes. This observation suggests that cell cycle regulation is perturbed in IVM derived oocytes as small foci are involved in the cell cycle regulatory process [52–53]. In contrast to *in vivo* matured oocytes, mock injected IVM oocytes had a higher number of large γ-H2AX foci which was significantly higher than ICSI control.

Since the plasma membrane of the *in vitro* matured oocytes are less elastic than those matured *in vivo* [56], it is possible that the process of needle insertion and ooplasmic aspiration might have influenced the results in the study. On the other hand, these mechanical aspects in ICSI had no effect on blastocyst formation in bovine oocytes [57]. Though, ICSI was performed by a single individual and the differences in the process of needle penetration, ooplasm leakage and subsequent degeneration was not significantly different between *in vivo* and *in vitro* matured group, it is still possible that the ultra-structural changes in IVM oocytes are responsible for immediate stress response and genetic instability observed in this study.

Although the results of the study are of interest, and have been understudied in the last decade by the ART field, the experimental design contains certain limitations. The use of the mouse as a model for human ICSI cannot be compared since it is difficult to perform ICSI successfully in this species using the clinically applied methods. Numerous studies show good success rates with ICSI in the mouse when used piezo injectors [23, 44] which were not used in the present set up. Secondly, there is no data on the developmental competence of the mock injected oocytes beyond PN stage which is very important to address the impact of H2AX phosphorylation on the genetic instability at later stages of preimplantation development. However, it has been shown that haploid parthenotes exhibit poor development and increased apoptosis due to genomic instability [58] which made us exclude this parameter in the present experimental setup. Another limitation is the influence of PVP on the oocytes which was not elucidated in the present manuscript. There is possibility that some amount of PVP is entering the oocyte during the process of mock injection, and importantly earlier study has shown that *in situ* DNA fragmentation can occur when poor quality spermatozoa are exposed to PVP in ICSI dish [20]. Hence further studies are required to rule out these confounding factors on our results.

In conclusion, there is scarce information about the genetic and functional changes that occurs in oocytes subjected to ICSI. Hence the results presented in this study may have some exciting clinical implications. Though, the experimental model and technique used in the present study did not mimic clinical ICSI completely, our results could be integrated in understanding of ICSI practice to optimize the conditions especially when more vulnerable oocytes are used.

## Supporting Information

S1 Dataset Raw data of ROS intensity in superovulated oocytes, IVM oocytes, mitochondrial distribution pattern in in vivo matured and IVM oocytes, H2AX foci in in vivo matured and IVM oocytes. (XLSX)
